# Thioredoxin 80-Activated-Monocytes (TAMs) Inhibit the Replication of Intracellular Pathogens

**DOI:** 10.1371/journal.pone.0016960

**Published:** 2011-02-18

**Authors:** Ximena Cortes-Bratti, Eugénie Bassères, Fabiola Herrera-Rodriguez, Silvia Botero-Kleiven, Giuseppe Coppotelli, Jens B. Andersen, Maria G. Masucci, Arne Holmgren, Esteban Chaves-Olarte, Teresa Frisan, Javier Avila-Cariño

**Affiliations:** 1 Department of Cell and Molecular Biology, Karolinska Institutet, Stockholm, Sweden; 2 Centro de Investigación en Enfermedades Tropicales, Facultad de Microbiología Universidad de Costa Rica, San José, Costa Rica; 3 Swedish Institute for Infectious Disease Control, Solna, Sweden; 4 Department of Microbiology and Risk Assessment, National Food Institute, Soeborg, Denmark; 5 Department of Biochemistry and Biophysics, Karolinska Institutet, Stockholm, Sweden; Duke University Medical Center, United States of America

## Abstract

**Background:**

Thioredoxin 80 (Trx80) is an 80 amino acid natural cleavage product of Trx, produced primarily by monocytes. Trx80 induces differentiation of human monocytes into a novel cell type, named Trx80-activated-monocytes (TAMs).

**Principal Findings:**

In this investigation we present evidence for a role of TAMs in the control of intracellular bacterial infections. As model pathogens we have chosen *Listeria monocytogenes* and *Brucella abortus* which replicate in the cytosol and the endoplasmic reticulum respectively. Our data indicate that TAMs efficiently inhibit intracellular growth of both *L. monocytogenes* and *B. abortus*. Further analysis shows that Trx80 activation prevents the escape of GFP-tagged *L. monocytogenes* into the cytosol, and induces accumulation of the bacteria within the lysosomes. Inhibition of the lysosomal activity by chloroquine treatment resulted in higher replication of bacteria in TAMs compared to that observed in control cells 24 h post-infection, indicating that TAMs kill bacteria by preventing their escape from the endosomal compartments, which progress into a highly degradative phagolysosome.

**Significance:**

Our results show that Trx80 potentiates the bactericidal activities of professional phagocytes, and contributes to the first line of defense against intracellular bacteria.

## Introduction

Monocytes/macrophages constitute one of the first lines of defense of the innate immune response against infectious agents. These cells are dedicated to the elimination of infectious microbes by phagocytosis. Newly formed phagosome containing bacteria will mature along the endocytic pathway, which culminates in a highly degradative phagolysosome through fusion with lysosomes [1]. The maturation process of phagosomes is tightly regulated, but many intracellular pathogens have developed sophisticated ways to circumvent phagosomes maturation in order to avoid destruction and ultimately multiply inside these cells. Such strategies include rapid escape from the phagosome to access either: i) the cytosol (*Listeria monocytogenes*); or ii) the endoplasmic reticulum (*Brucella abortus*); and iii) arresting the normal maturation of the phagosome (*Mycobacterium tuberculosis*) [2], [3], [4], [5].

Identification of host factors that may regulate survival of pathogens within macrophages has strong implication for developing novel therapeutic approaches. In this context, it is interesting to study the biological properties of thioredoxin (Trx) 80, a truncated form of Thioredoxin (Trx). Trx is a major intracellular protein-disulfide reductase [6]. Trx80 is an 80 amino acid natural cleavage product of Trx, produced primarily by monocytes [7] that was initially described as an eosinophilic cytotoxicity enhancing factor (ECEF) [8]. Trx80 lacks the c-terminal βα motif of the thioredoxin fold [9], [10] and it is devoid of the disulfide oxidoreductase activity of thioredoxin. Recombinant Trx80 evokes proliferation of normal human peripheral blood mononuclear cells and acts as a chemokine for monocytes, T cells and polymorphonuclear cells [11].

We have previously demonstrated that Trx80 triggers innate immunity by inducing the differentiation of human monocytes into a novel cell type, which we have named **T**rx80-**a**ctivated-**m**onocytes (TAMs). Trx80 activates monocytes and induces: i) up-regulation of cell surface pathogen recognition receptors, such as CD14 (receptor with a broad substrate specificity, stimulates phagocytosis of microbes and inhibits apoptosis), CD1a (associated to the recognition of lipids and glycolipids by NK and T-cells) and Mannose Receptor (binds certain carbohydrate moieties present on the surface of bacteria); ii) up-regulation of molecules essential for T-cell activation and function, such as CD86, CD54, CD40; iii) high pinocytic capacity; iv) significantly higher release of the pro-inflammatory cytokines TNFα, IL-1β and IL-6. Interestingly, Trx80 is the first bona fide cytokine shown to induce production of the regulatory and anti-inflammatory cytokines IL-12 and IL-10, respectively [12], [13], [14]. In this investigation we demonstrate that Trx80-activated monocytes are able to control more efficiently the replication of intracellular bacteria by preventing their escape from the endosomal compartments that will further mature into a highly degradative phagolysosome.

## Materials and Methods

### Reagents

Recombinant Trx80 was prepared as described in detail earlier [15]. Briefly, this protein was over-expressed in *E. coli*, where it was found in inclusion bodies. Subsequently, Trx80 was solubilized from these bodies utilizing urea and the resulting solution dialyzed and subjected to ion-exchange and reverse-phase chromatography, followed by gel filtration through a Sephadex G-75 column. The purity of the final preparation was confirmed by SDS-polyacrylamide gel electrophoresis. Finally, in order to eliminate contamination by endotoxin, these Trx80 preparations were first passed through a polymyxin B column and then in a positively charged sterile filter. The endotoxin content was less than 0.2 ng/ml in cell cultures exposed to 100 nM Trx80 as determined by the Limulus amoebocyte lysate assay (Charles River Endosafe Inc., Charleston, S.C., USA). Stock solutions were maintained at −20° C until use. RPMI 1640 cell culture medium, L-glutamine, penicillin and streptomycin (Gibco, BRL, Life technologies, Paisley, Scotland); fetal calf serum (FCS, Hyclone, Logan UT); LymphoprepTM (Axis-Shield PoC AS, Norway); trypan blue, chloroquine, gentamicin solution, paraformaldehyde solution and 6-well ultralow cell culture attachment plates (Sigma, Chemical, St. Louis, MO); CD14+ human microbeads (Miltenyi Biotec, Friedrich-Ebert-Straβe, Germany); Lysotracker Red DND 99 and 2′7′-dichlorofluorescein diacetate (H2DCFDA, Molecular probes); Vecta Shield mounting medium (Burlingame, CA); the FITC mouse monoclonal antibodies anti-CD14 (APC; M
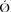
P9) and the isotype antibody IgG2a (Becton Dickinson, San Diego, CA); for bacterial culture media, brain heart infusion (BHI) broth, Luria Bertani (LB) broth, and Bacto-Agar (Difco, France).

### Purification of CD14+ cells

Human peripheral blood mononuclear cells (PBMC) were prepared from the blood of healthy donors (Blood Bank, Karolinska University Hospital) by the standard procedure of Ficoll-Paque centrifugation, and viability was assessed by trypan blue exclusion. Thereafter, monocytes were isolated using the CD14+ MACS procedure, according to the manufacturer's instructions and as previously described [14]. Purified CD14+ cells were suspended in complete RPMI 1640 medium containing 10% heat-inactivated FCS, 2 mM L-glutamine, 100 U/ml penicillin and 100 micrograms/ml streptomycin (complete medium). Cells were seeded in 6-well ultralow attachment plates, previously warmed at 37°C with 2 ml RPMI without serum. The use of human buffy coats was approved by the ethics committee of the Karolinska Institutet.

### Culture conditions and treatments

Following resuspension in complete medium at a concentration of 10^6^ cells per ml, CD14+ cells were cultured in the absence or presence of 100 nM Trx80. All cells were cultured in a final volume of 5–8 ml on 6-well ultralow attachment cell culture plates, at 37°C with 5% CO_2_. For the inhibition of lysosomal enzymes, chloroquine (10 µM) was added during the infection and maintained throughout the experiment.

### Flow cytometry analysis

Cell cultures were analyzed for CD14+ expression after 24 h and 48 h in culture. Approximately, 5×10^5^ cells were incubated with normal human serum for 30 minutes on ice to block Fc receptors and reduce the non-specific binding of the monoclonal antibodies (mAbs). The cells were incubated with FITC-conjugated CD14 mAb or the isotype-matched control IgG2a for 45 minutes on ice, washed once with 1 ml PBS and suspended in 500 µL of the same buffer. Thereafter, 10^4^ cells were acquired on a FACSCalibur flow cytometer (Becton Dickinson) and analyzed using the CellQuest software (Becton Dickinson). As previously described [14], the cell characteristic forward-scatter pattern was used to generate a gate and only this population was analyzed.

### Bacterial strains

The bacterial strains used were: *Brucella abortus* 2308, *Listeria monocytogenes* strain EGD (BUG600, serotype 1/2a, kindly provided by Dr. Martin E. Rottenberg), *L. monocytogenes* EGDe harbouring the plasmid GFP-expressing pNF8 plasmid [16], *L. monocytogenes* NF-L327, containing a transcriptional gene fusion *actA-gfp-plcB*, that expresses GFP only when bacteria escape in the cytosol [17] (kindly provided by Dr. Nancy E. Freitag). The *Brucella* strain was routinely grown in LB medium, and the *Listeria* strains were grown in BHI medium.

### Monocyte infection and colony forming units (CFU) assay

CD14+ control monocytes and thioredoxin activated monocytes (TAMs) were incubated with *L. monocytogenes* or *B. abortus* for 30 and 45 minutes, respectively at 37°C, 5% CO_2_. Cells were further incubated for 1 hour in medium containing gentamicin 100 µg/ml for elimination of extracellular bacteria (bacteria uptake). Cells were washed once and maintained in RPMI containing 5 µg/ml of gentamicin to prevent extracellular bacterial growth and re-infection. After the infection, samples were taken at different time points, cells were counted by trypan blue exclusion, washed once with PBS and lysed with 0.1% Triton X-100 in PBS. Lysates were prepared from 3×10^5^ viable cells, and 20 microliters aliquots were plated on LB or BHI agar plates. Plates were incubated for 48 hours at 37°C and colony forming units (CFU) were quantified. *Listeria* strains were used at a multiplicity of infection (MOI) of 25∶1 or 50∶1, and the *Brucella* experiments were performed at MOI of 100∶1.

### Immunofluorescence for detection of *B. abortus* internalization

Extracellular bacteria were detected using a cow anti-*Brucella* FITC conjugated antibody (1∶150), added to 200 µl of infected cells and incubated 30 minutes at 4°C under agitation. Cells were washed with 1 ml PBS, centrifuged, resuspended in 200 µl PBS, and transferred to slides to dry overnight at 37°C. Cells were then fixed in 4% paraformaldehyde for 10 minutes at 22°C, and incubated with ammonium chloride (50 mM) 10 minutes at 22°C, blocked for 30 minutes at 22°C with human plasma and permeabilized with 0.1% Triton X100 in PBS for 10 minutes. Rabbit anti-*Brucella* antibodies diluted 1∶200 in PBS containing 0.5% albumin were added and after incubation the slides were washed twice with PBS and once with 0.1% Triton X100 in PBS. The cells were incubated for 30 minutes with an Alexa 568-conjugated anti-rabbit secondary antibody, diluted 1∶1000 in PBS containing 0.5% albumin. This step allows to discriminate between extracellular bacteria (labelled with both FITC and Alexa 568-conjugated antibodies) and the intracellular bacteria (labelled only with the Alexa 568-conjugated antibody). Slides were washed as in the previous step. Nuclei were counterstained with DAPI and mounted in glass slides with VectaShield mounting medium (H1200). Slides were analyzed with a Leica DMRXA fluorescence microscope with a CCD camera (Hammamatsu) and images were captured with Improvision Openlab v.2 software.

### LysoTracker staining

The acidification of *Listeria* containing phagosomes was determined with the lysosomotropic agent LysoTracker red DND-99 following the manufacturer's instructions. After gentamicin incubation LysoTracker red DND-99 was added to a final concentration of 200 µM and maintained in the medium for the indicated time. In non infected cells or cells infected with the GFP-tagged *Listeria* were harvested and centrifuged at 2500 rpm for 5 minutes, washed 3 times with PBS 10% FBS (pre-warmed at 37°C) and diluted to a final concentration of 8×10^4^–10×10^4^ cells/ml. Aliquots of 70 µl were taken in triplicate, and spun at 200 rpm for 4 minutes (Cytospin 3, Shandon). Slides were fixed with freshly prepared 4% paraformaldehyde for 15 minutes at 22°C, and mounted in glass slides with VectaShield mounting medium (H1200). Cover slides were analyzed by fluorescence microscopy (Leica DMRXA fluorescence microscope). Colocalization of GFP-labeled bacteria within lysotracker positive vesicles was assessed in a minimum of 200 cells, from three independent slides by visual analysis, and by correlation of the GFP and Lysotracker Red fluorescence intensities using the Volocity software package (Perkin Elmer). To detect the efficiency of bacterial escape from the phagosome, cells infected with the *L. monocytogenes* strain NF-L327 were spun and fixed as previously described. Slides were analyzed by fluorescence microscopy (Leica DMRXA fluorescence microscope).

## Results

### TAMs efficiently control intracellular replication of *Listeria monocytogenes*

**Figure 1:**
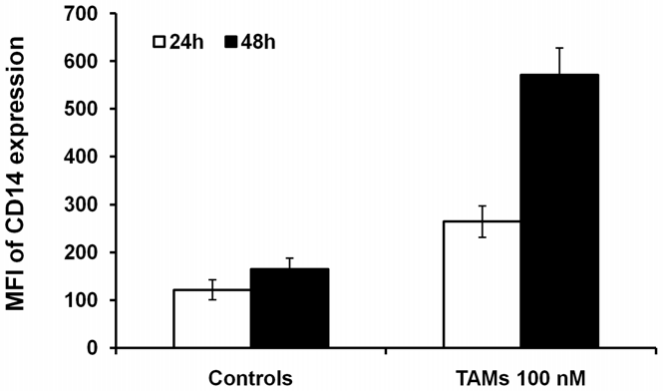
TAMs express high levels of CD14. Non-stimulated monocytes and TAMs generated with Trx80 at 100 nM were analyzed for CD14 expression after 24 and 48 hours in culture. MFI: mean fluorescence intensity (mean ± SEM of six independent experiments).

**Figure 2:**
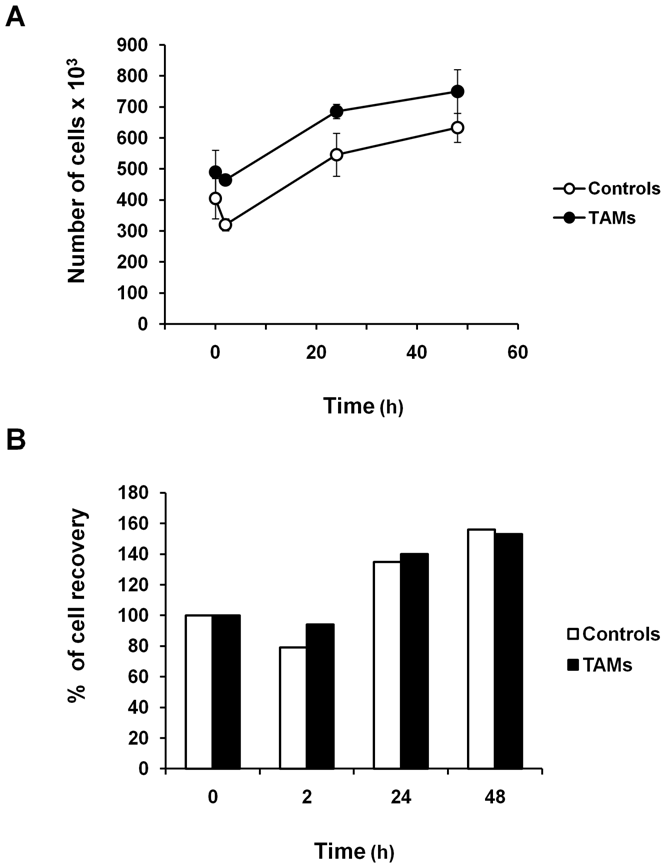
Growth curve of untreated control monocytes and TAMs after *L. monocytogenes* infection. Cells were infected as described in Materials and Methods. After the infection, samples were taken at the indicated periods of time, and number of viable cells was assayed by trypan blue exclusion. The data are presented as number of viable cells (panel A) and as percentage of cell recovery, being 100% of recovery at time 0 (panel B). Mean ± SEM of six independent experiments.

To assess whether Trx-80-activated monocytes contribute to control replication of intracellular pathogens, we have chosen as model *L. monocytogenes*, a bacterium that replicates in the cytosol of infected cells. As previously demonstrated [12], [13], [14], TAMs exhibited a 2- to 3-fold increase in the mean fluorescence intensity for the surface marker CD14 after 24 and 48 hours stimulation with 100 nM Trx80 compared to non-stimulated cells cultured in medium alone (Figure 1). Therefore, the subsequent experiments were performed with monocytes exposed to 100 nM Trx80 for 24 hours, and the levels of CD14 expression were routinely assessed to confirm the efficacy of activation. Growth curve kinetics demonstrated that the recovery of viable cells in infected control monocytes or TAMs were similar (Figures 2A and 2B).

**Figure 3:**
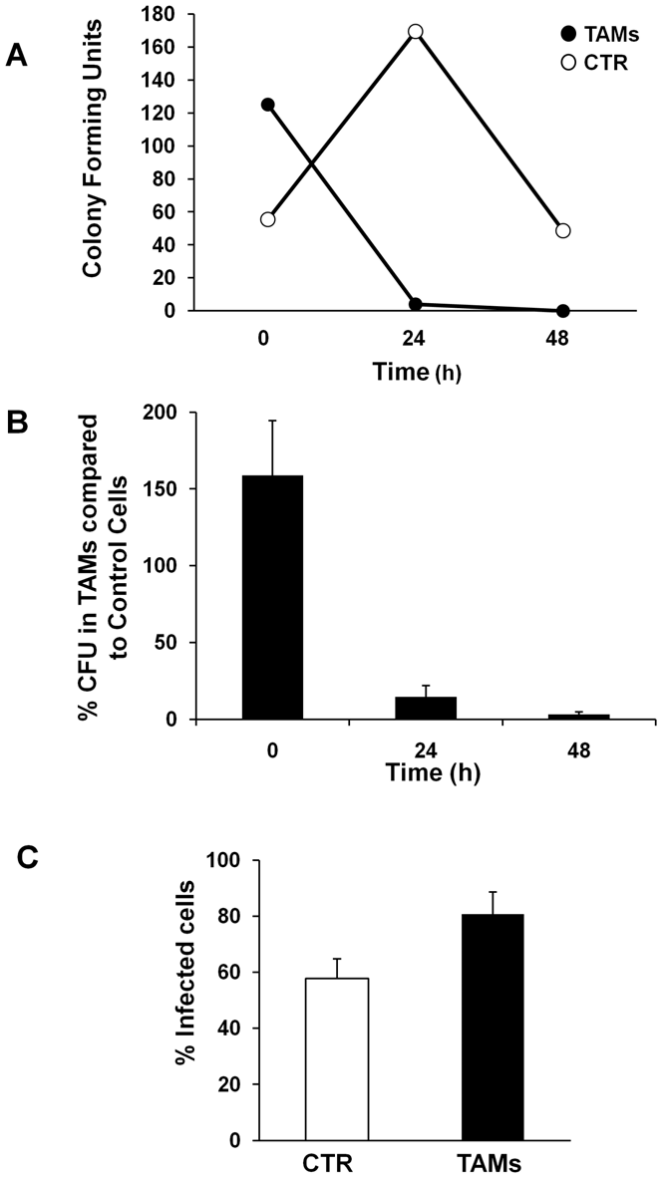
TAMs control replication of *Listeria monocytogenes.* **A**. Intracellular growth curve of *L. monocytogenes*. Cells were infected for 30 minutes at 37°C with a multiplicity of infection of 25∶1 bacteria per cell, and medium containing 100 µg/ml gentamicin was added for 1 h to kill extracellular bacteria (0 h). Cells were washed and RPMI supplemented with 5 µg/ml gentamicin was added. At the indicated periods of time, 3×10^5^ cells were lysed and colony forming units CFU were quantified, as described in Materials and Methods. A representative experiment is shown. **B**. Summary of the growth curves of *L. monocytogenes*. The data are presented as percentage of the number of CFU recovered in TAMs relative to the number of CFU recovered in control monocytes (mean ± SEM of five independent experiments). **C**. Cells were infected with *L. monocytogenes* for 30 minutes at 37°C with a MOI 25∶1 and medium containing 100 µg/ml gentamicin was added for 1 h to kill extracellular bacteria. Intracellular bacteria were detected by immunofluorescence analysis as described in Materials and Methods (mean ± SEM of five independent experiments).

Time kinetic experiments demonstrated that TAMs limit bacterial replication much more efficiently than control cells 24 hours post-infection, since the number of viable bacteria in TAMs was less than 5% of that observed in control cells (Figure 3A). The number of bacteria in TAMs remained significantly reduced up to 48 hours post-infection (Figures 3A and 3B). We next investigated whether the difference in bacterial recovery was due to reduced entry in TAMs compared to control monocytes. To assess bacterial uptake, control monocytes and TAMs were infected with *L. monocytogenes* at MOI 25 for 30 minutes, and 100 µg/ml gentamicin was added for 1 hour to kill the non-internalized bacteria. The rate of uptake of *L. monocytogenes* was higher in TAMs compared to control cells, as assessed by gentamicin assay (Figure 3B) or immunofluorescence analysis (Figure 3C).

### TAMs prevent the escape of *L. monocytogenes* from the phagosome

**Figure 4:**
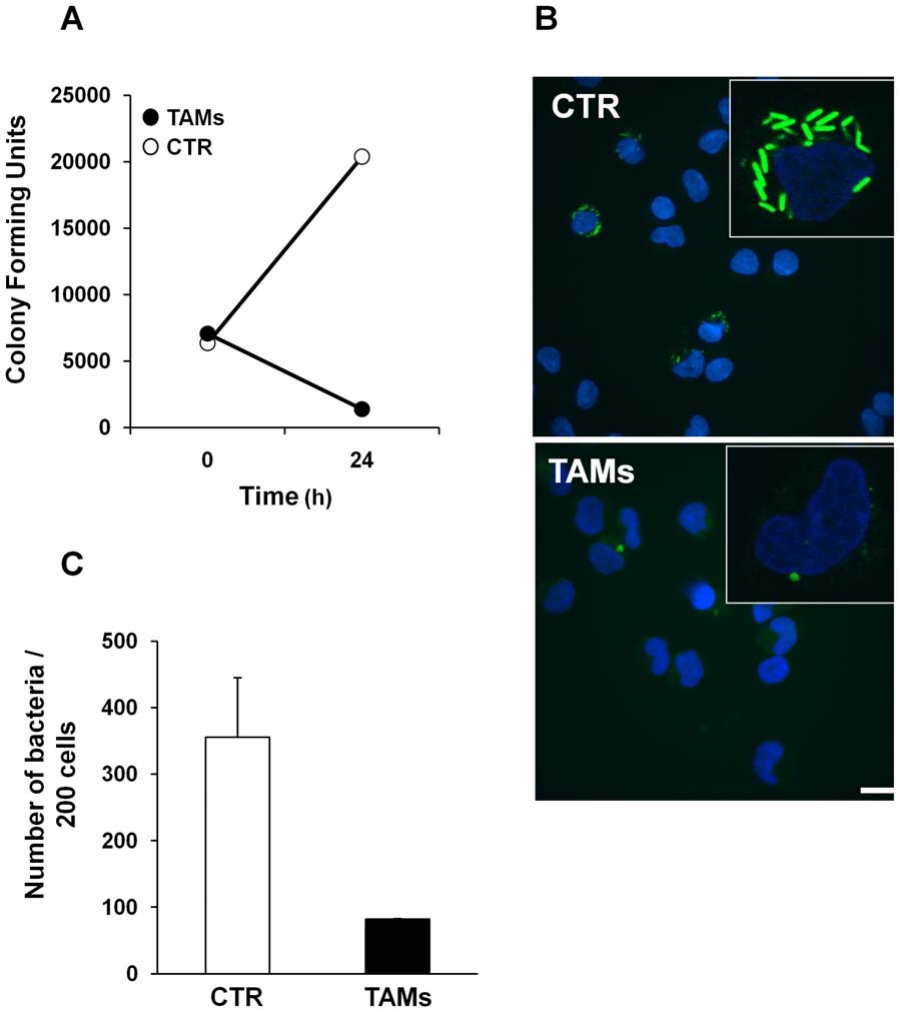
Escape of *L. monocytogenes* NF-L327 into the cytosol of infected monocytes. **A**. Cells were infected with the *L. monocytogenes* strain NF-L327, that expresses GFP when translocated into the cytosol, at MOI 50∶1 for 30 minutes and medium containing 100 µg/ml gentamicin was added for 1 h to kill extracellular bacteria (0 h). Cells were washed and maintained in RPMI supplemented with 5 µg/ml gentamicin. Cells were lysed after infection (0 h) and 24 h in culture and the colony forming units CFU were quantified, as described in Figure 3A. A representative experiment is shown. **B**. Cells were infected with the *L. monocytogenes* NF-L327 at MOI 50∶1 as described in A and samples were taken 8 hours post-infection. Cytospin and fluorescence imaging were performed as described in Materials and Methods. Bar represents 5 µm. **C**. Quantification of GFP-labeled *Listeria* was performed using the Volocity software. The data are presented as number of bacteria in 200 cells (SEM of two independent experiments).

We next investigated whether the lower level of bacterial replication in TAMs was associated with reduced escape of *L. monocytogenes* from the phagosome into the cytosol of infected cells. As tool we used the NF-L327 strain, which expresses GFP upon bacterial release into the cytosol [17]. Growth curves indicated that uptake of the NF-L327 strain was similar in TAMs and control cells, while a lower recovery of viable bacteria was detected 24 h after infection in Trx80-activated monocytes (Figure 4A). These data correlated with the immunofluorescence analysis that showed a 3- to 4-fold increase in the number of GFP-positive bacteria in non-stimulated monocytes compared to that observed in TAMs 8 hours after infection (Figures 4B and 4C), indicating that *L. monocytogenes* NF-L327 was efficiently internalized and trapped in the phago-lysosome upon Trx80-activation.

### TAMs enhance *L. monocytogenes* compartmentalization within acidified vesicles

**Figure 5:**
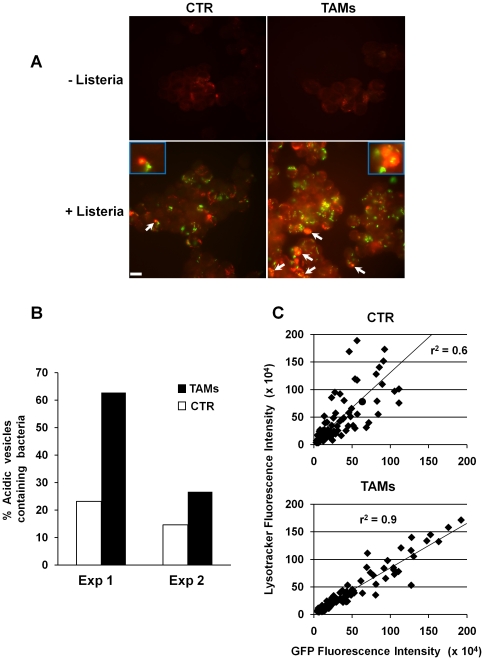
Localization of GFP-tagged *L. monocytogenes* in acidic compartments. **A**. Cells were infected with the GFP-tagged *L. monocytogenes* at MOI 50∶1 for 4 hours, and treated with the acidotropic dye Lysotracker Red. Cytospin centrifugation was performed and cells were fixed with 4% paraformaldehyde. Lysotracker Red positive vesicles containing GFP-labeled bacteria are indicated with arrows. Bar represents 5 µm. **B**. The percentage of Lysotracker Red positive vesicles containing GFP-labeled bacteria was quantified in two independent experiments. A minimum of 200 cells were analyzed. **C**. Scatter plot analysis showing the correlation between the GFP and Lysotracker Red fluorescence intensities in control cells and TAMs quantified by the Volocity software. A correlation coefficient value of r^2^ equal to 1 indicates 100% bacterial localization with the lysosomal compartments.

The data presented in Figures 3 and 4 indicate that TAMs internalize *L. monocytogenes* efficiently, prevent its escape into the cytosol and exhibit enhanced bactericidal activity compared to the non-stimulated cells. Since lysosomes represent one of the best-characterized vesicles where activated monocytes exert their bactericidal activity, we assessed whether Trx80 stimulation was associated with an enhanced trapping of bacteria within the lysosomal compartment, thus preventing escape into the cytosol. Monocytes and TAMs were infected with a GFP-tagged *Listeria* at MOI 50, and localization of the bacteria was assessed 4 hours post-infection, using Lysotracker Red to visualize acidified vacuoles. Uninfected control cells and TAMs presented similar levels of lysosomal labeling (Figure 5A). In both cell types, infection was associated with an increased number of Lysotracker Red positive vesicles (Figures 5A and 5B). A 2- to 3-fold increase in the number of bacteria within acidified compartments was observed in TAMs, compared to non-stimulated monocytes 4 hours post-infection (Figure 5B). The Volocity software was further used to evaluate the co-localization between the GFP and the Lysotracker Red fluorescence intensities. A correlation coefficient value of r^2^ equal to 1 indicates 100% of bacterial localization with the lysosomal compartments; Figure 5C shows that TAMs have an r^2^ value of 0.9, while this is reduced to 0.6 in control cells. Collectively, these results indicate that Trx80 activation retains *L. monocytogenes* within acidified compartments and prevents bacterial escape and replication in the cytosol.

### Trx80-activated monocytes kill *Listeria* in the lysosomal compartments

**Figure 6:**
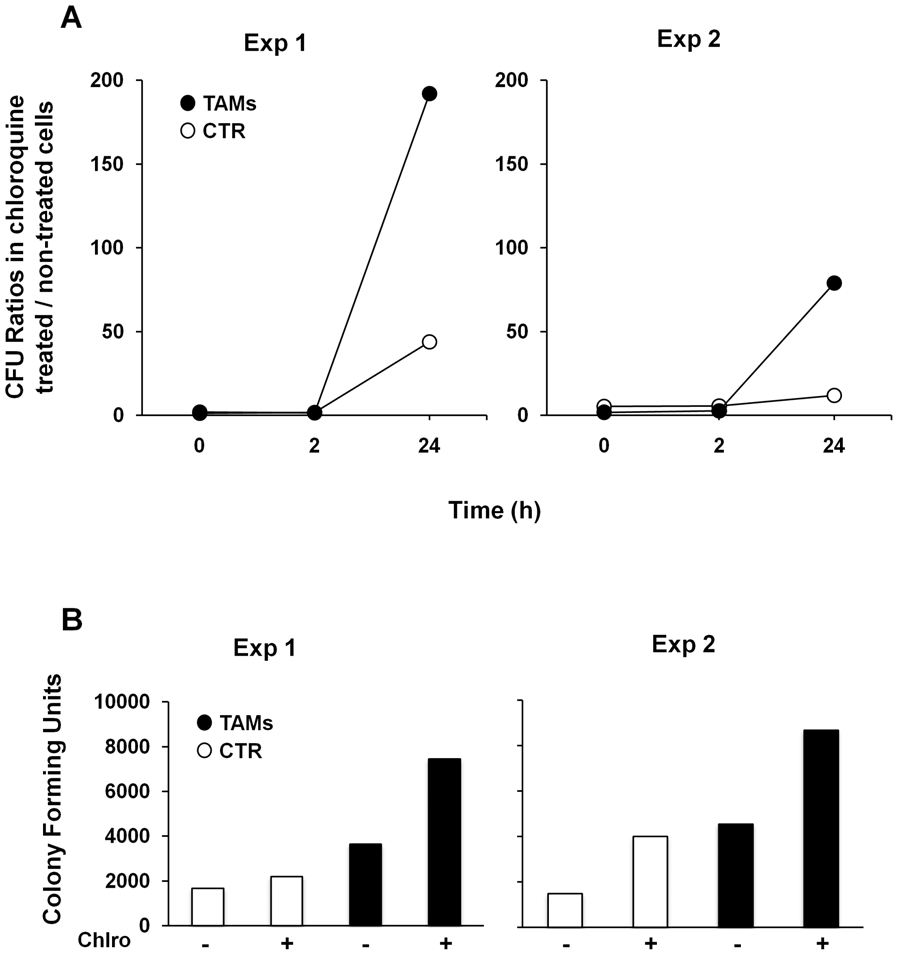
Increased recovery of *L. monocytogenes* in cells treated with the lysosomotrophic agent chloroquine. Control monocytes and TAMs were infected with *L. monocytogenes* EGD strain at MOI 25∶1 for 30 minutes in the absence or presence of chloroquine (10 µM) and the treatment was maintained throughout the experiment. **A**. At the indicated periods of time, cells were lysed and the CFU quantified as described in Figure 3A. Results are shown as ratio between the number of CFU recovered in chloroquine treated cells and the number of CFU recovered in non-treated cells at each time point. **B**. Number of CFU at time 0 h.

To investigate the role of lysosomal enzymes in TAM-induced bactericidal activity on *L. monocytogenes*, we compared the effect of chloroquine (10 µM) on bacterial recovery at 0 (uptake), 2 and 24 hours post-infection by gentamicin assays. Chloroquine treatment enhanced the recovery of viable bacteria 24 hours after infection in both cell types. However, the amount of viable bacteria recovered in chloroquine treated TAMs was significantly higher than that observed in non stimulated cells (Figure 6A). Chloroquine treatment did not inhibit bacterial entry in control cells or TAMs. On the contrary, we have observed an increased recovery of *L. monocytogenes* in both cell types at time 0 (Figure 6B). These data indicate that lysosomal-dependent degradation is a key bactericidal mechanism induced by Trx-80 stimulation.

### TAMs efficiently control intracellular replication of *Brucella abortus*

**Figure 7:**
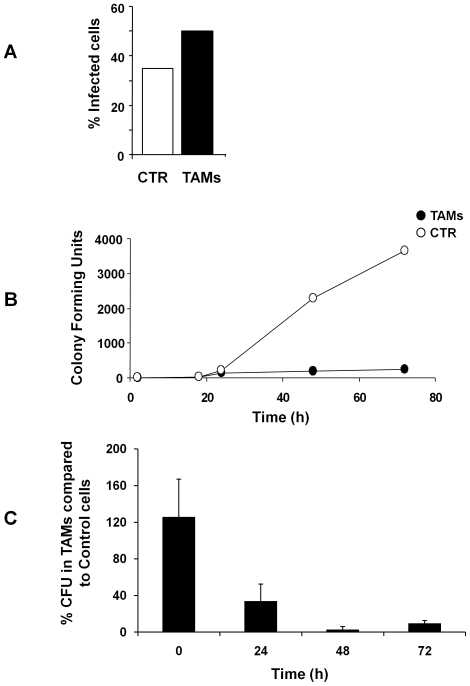
TAMs control the replication of *Brucella abortus*. **A**. Cells were infected with *B. abortus* for 45 minutes at 37°C at a MOI 100∶1 and medium containing 100 µg/ml gentamicin was added for 1 hour to kill extracellular bacteria. Intracellular bacteria were detected by immunofluorescence analysis as described in Materials and Methods. **B**. Intracellular growth curve of *B. abortus*. Cells were infected 45 minutes at 37°C at a MOI 100∶1 and medium containing 100 µg/ml gentamicin was added for 1 h to kill extracellular bacteria (0 h). Cells were washed and RPMI supplemented with 5 µg/ml gentamicin was added. At the indicated periods of time, cells were lysed and the CFU quantified as described in Figure 3A. A representative experiment is shown. **C**. Summary of growth curves of *B. abortus*. The data are presented as percentage of the number of CFU in TAMs relative to the number of CFU in control monocytes (SEM of three independent experiments).

We next assessed whether the effect of Trx80 on replication of intracellular pathogens was dependent on the site of bacterial replication, and we therefore have investigated the ability of TAMs to control infection with *Brucella abortus*, a Gram negative bacterium that replicates in the ER of infected cells. Control monocytes and TAMs were infected with *B. abortus* at MOI 100∶1 for 45 minutes, and 100 µg/ml gentamicin was added for 1 hour to kill the non-internalized bacteria. As shown in Figure 7A, the rate of uptake of *B. abortus* was more efficient in TAMs compared to non-stimulated cells, as assessed by immunofluorescence analysis. For long time kinetics, infected cells were maintained in medium containing 5 µg/ml gentamicin, lysed at the indicated times and the number of colony forming units (CFUs) was evaluated after incubation for 48–72 hours at 37°C. The number of viable bacteria was very low in the initial phase of the infection in both cell types (Figure 7B). In control cells, the recovery of bacteria steadily increased in a time-dependent manner up to 72 hours post-infection, while a significantly lower number of CFUs was detected in TAMs at 24 hours post-infection, and this was maintained up to 72 hours (Figures 7B and 7C).

Collectively these data indicate that Trx80 activation of monocytes enhances their bactericidal activities and exerts an efficient control of the infection by intracellular bacteria, independently on the site of replication.

## Discussion

Our findings shed new light on the role of thioredoxin (Trx) 80 as an effector molecule of the innate immune response against intracellular pathogens. We have previously demonstrated that Trx80 stimulates human monocytes to differentiate into a novel cell type, Trx80-Activated-Monocytes (TAMs). TAMs have higher pinocytic capacity, and enhanced secretion of pro-inflammatory cytokines [14]. As these characteristics resemble those of activated macrophages, we suggested that TAMs may play a major role in the defense against intracellular pathogens [12], [13], [14]. In this study, we performed a set of experiments to test this hypothesis. We chose to focus on two intracellular pathogens known to survive and replicate inside monocytes: *Brucella abortus* and *Listeria monocytogenes.* To achieve their goal, they must interfere with the different stages of phagosomal maturation to prevent their killing. Phagocytosis of *Brucella* and *Listeria* is mediated by scavenger receptors and functional lipid rafts [18], [19]. Our findings show that TAMs internalize *Listeria* with higher efficiency than non-stimulated monocytes, since a higher number of bacteria was recovered from infected TAMs after the internalization step, as assessed by gentamicin assays and immunofluorescence analysis (Figure 3). This is in line with the enhanced phagocytic capacity of TAMs [14]. Time kinetics of infection indicate that TAMs limit *Brucella* and *Listeria* replication during the early/intermediate stages of infection (Figures 3 and 6), which coincide with the intermediate/late stages of phagosome maturation [1], neutralizing the strategies used by *Brucella* and *Listeria* to avoid phagolysosome-dependent degradation.

*Listeria* escapes the phagosome before it matures and fuses with the lysosome. This step is very rapid, and occurs within five minutes from the bacterial engulfment into the host cell, and it is mediated by the production and secretion of listeriolysin O (LLO) and two phospholipases, PI-PLC and PC-PLC. These enzymes cause the breakdown of the membrane of the *Listeria*-containing phagosome and thereby enable the bacteria to escape to the cytosol where they replicate [1]. The data presented in Figures 4 and 5 indicate that more bacteria are localized within acidified vesicles 4 hours after infection, resulting in 3- to 4- fold decreased recovery of *Listeria* in the cytosol of infected cells. Trx80-dependent activation may alter the environment of the phagosome preventing activation of LLO and secretion of phospholipases. Maturation of the phagolysosome *per se* may be accelerated upon cell activation and bacteria can be killed with a faster kinetic. Alternatively, Trx80 activation may enhance the autophagy process, resulting in formation of autophagosomes [20]. Similar results have been reported in macrophages activated with IFN-γ or LPS [21], indicating that at least for the control of *Listeria* replication, Trx80 activation has similar effects as other well-known activators of the macrophage function. It has been previously shown that reduction of LLO is required to activate its lytic activity and this is achieved by the enzyme γ-interferon-inducible lysosomal thiol reductase (GILT, also called Ifi30) [22]. It is unlikely that Trx80 contributes to this process, since it lacks the disulfide oxidoreductase activity and Trx80 stimulation of monocytes in absence of T lymphocytes is not sufficient to induce IFN–γ production and secretion [12].

Survival of *Brucella* inside the cell relies on avoiding fusion of the *Brucella*-containing vacuole (BCV) with lysosomes. For its intracellular survival, *Brucella* produces cyclic glucans and requires the type IV secretion system VirB. Cyclic glucans modulate maturation of BCV to avoid fusion with lysosomes [23], while VirB is required for the late BCV maturation events corresponding to sustained interaction and fusion with the ER [4]. TAMs could act either by neutralizing the cyclic glucans at the early stage of the infection or interfering with the VirB system to prevent fusion of the BCV with the ER.

The last step in phagosomal maturation is the fusion of the phagosome with the lysosome that culminates in the formation of the phagolysosome [1]. Our experiments show a higher recovery of viable bacteria in chloroquine-treated TAMs 24h post-infection compared to chloroquine treated non-stimulated cells (Figure 6). These data are in agreement with the results presented in Figures 3 and 4, which showed a higher uptake of bacteria and an enhanced localization of the GFP-tagged *L. monocytogenes* in acidic compartments as early as 4 hours after infection in TAMs, and indicate that lysosomal degradation is one of the main bactericidal mechanisms induced by Trx-80 activation in monocytes.

Many pathogens have evolved several strategies to escape the host's innate and adaptive immune responses [24]. Therefore it would not be surprising if bacteria could overcome the Trx80-induced monocyte activation. It was recently reported that the *Salmonella* type III secretion factor (SIrP) is an E3 ubiquitin ligase that can mediate ubiquitination of human thioredoxin, reducing the levels of Trx and rendering the cells more prone to cell death [25]. The strategy of bacteria like *Salmonella* to decrease the levels of Trx will directly affect the production of Trx80, and consequently impair the TAMs-mediated immune response.

The production of Trx80 *in vivo* is not fully understood; however, several reports indicate that activated monocytes are the main producers of Trx80 [26], [27], [28]. The most likely mechanism is that full-length Trx is cleaved at the protein level by an inducible protease to generate Trx80. Isolated monocytes from healthy donors cleave exogenously added Trx to the truncated form within 1 min after addition of full-length Trx. Upon cleavage of Trx, the truncated species adhere to the membrane of macrophages [29]. *In vitro* established T, B and monocytic cell lines produce Trx80, whereas isolated T and B lymphocytes and monocytes produce Trx80 in minimal amounts. However, when PBMC or isolated monocytes are exposed to cytokines (IFN-γ, IL-1α), bacterial products (fMLP, LPS, ionomycin) or mitogens *(*Phorbol myristate acetate, Concanavalin A, pokeweed mitogen, phytohemagglutinin) the production of Trx80 is increased [26], [27], [28].

Does production of Trx80 have a role *in vivo*? Up to now there are no data regarding the efficacy of this protein in controlling replication of intracellular bacteria *in vivo*. However, Trx80 is detected in plasma of healthy blood donors in concentration up to 20 nM [15]; and functional assays show increased levels of Trx80 in patients with severe schistosomiasis [8] and patients affected by chronic inflammatory conditions, such as rheumatoid arthritis [30]. These data indicate that Trx80 is produced in physiological conditions, and that its production/secretion is increased in pathologies associated with chronic inflammation. Local concentrations of Trx80 in health or disease (infection/inflammation) should be much higher than the reported in plasma, therefore our experimental settings (100 nM) should be within the range at the site of cell production. Thus, at the site of infection/inflammation, cells can be induced to produce full-length Trx and/or Trx80, whose chemoattractant properties could result in recruitment of polymorphonuclear neutrophils, T cells and monocytes [11], [28], [31]. These monocytes can cleave full-length Trx to Trx80, as well as produce their own Trx80 [28], [29]. This situation would then lead to activation of the monocytes by Trx80, up-regulating expression of pattern recognition receptors (CD1a, CD14, MR), receptors for communication with T or NK cells (CD40, CD54, CD86) and the release of cytokines and chemokines (TNFα, IL-1β, IL-6 and IL-8). Cross-talk between TAMs and T cells will stimulate the TAMs to produce IL-12, which in turn, will subsequently trigger the production of IFN-γ by the T lymphocytes [12], [13], [14].

In conclusion, we have demonstrated that Trx80 possesses an intrinsic capacity to control intracellular infections, and that TAMs are effective against bacteria, such as *B. abortus* and *L. monocytogenes*. The effects of Trx80 on monocytic cells are similar to those induced by IFN-γ, which plays a mandatory role in protection against intracellular pathogens. These data suggest that TAMs are efficient effectors of the innate immune response and represent a first line of defense against intracellular infections, before the immune system can mount a proper T-cell response.
